# miR-302a Inhibits Metastasis and Cetuximab Resistance in Colorectal Cancer by Targeting NFIB and CD44

**DOI:** 10.7150/thno.36605

**Published:** 2019-10-22

**Authors:** Lina Sun, Ying Fang, Xin Wang, Yanan Han, Feng Du, Cunxi Li, Huaying Hu, Hao Liu, Qi Liu, Jing Wang, Junrong Liang, Ping Chen, Hongbin Yang, Yongzhan Nie, Kaichun Wu, Daiming Fan, Robert J. Coffey, Yuanyuan Lu, Xiaodi Zhao, Xin Wang

**Affiliations:** 1 The Affiliated Children's Hospital of Xi'an Jiaotong University, Xi'an 710003, China.; 2 State Key Laboratory of Cancer Biology, National Clinical Research Center for Digestive Diseases and Xijing Hospital of Digestive Diseases, Fourth Military Medical University, Xi'an 710032, China.; 3 Department of Gastroenterology, Tangdu Hospital, Fourth Military Medical University, Xi'an 710038, China.; 4 Jiaen Genetics Laboratory, Beijing Jiaen Hospital, Beijing 100191, China.; 5 School of Medicine, Xiamen University, Xiamen 361002, China.; 6 Department of Biomedical Informatics and Center for Quantitative Sciences, Vanderbilt University Medical Center, Nashville, Tennessee 37232, USA.; 7 Department of Medicine, Vanderbilt University Medical Center, Nashville, Tennessee 37232, USA.; 8 Department of Veterans Affairs Medical Center, Nashville, Tennessee 37212, USA.; 9 National Institute of Biological Sciences, Beijing 102206, China.

**Keywords:** miRNA, colorectal cancer, metastasis, targeted therapy, drug resistance.

## Abstract

**Introduction**: Metastasis and drug resistance contribute substantially to the poor prognosis of colorectal cancer (CRC) patients. However, the epigenetic regulatory mechanisms by which CRC develops metastatic and drug-resistant characteristics remain unclear. This study aimed to investigate the role of miR-302a in the metastasis and molecular-targeted drug resistance of CRC and elucidate the underlying molecular mechanisms.

**Methods**: miR-302a expression in CRC cell lines and patient tissue microarrays was analyzed by qPCR and fluorescence* in situ* hybridization. The roles of miR-302a in metastasis and cetuximab (CTX) resistance were evaluated both *in vitro* and *in vivo*. Bioinformatic prediction algorithms and luciferase reporter assays were performed to identify the miR-302a binding regions in the NFIB and CD44 3'-UTRs. A chromatin immunoprecipitation assay was performed to examine NFIB occupancy in the ITGA6 promoter region. Immunoblotting was performed to identify the EGFR-mediated pathways altered by miR-302a.

**Results**: miR-302a expression was frequently reduced in CRC cells and tissues, especially in CTX-resistant cells and patient-derived xenografts. The decreased miR-302a levels correlated with poor overall CRC patient survival. miR-302a overexpression inhibited metastasis and restored CTX responsiveness in CRC cells, whereas miR-302a silencing exerted the opposite effects. NFIB and CD44 were identified as novel targets of miR-302a. miR-302a inhibited the metastasis-promoting effect of NFIB that physiologically activates ITGA6 transcription. miR-302a restored CTX responsiveness by suppressing CD44-induced cancer stem cell-like properties and EGFR-mediated MAPK and AKT signaling. These results are consistent with clinical observations indicating that miR-302a expression is inversely correlated with the expression of its targets in CRC specimens.

**Conclusions**: Our findings show that miR-302a acts as a multifaceted regulator of CRC metastasis and CTX resistance by targeting NFIB and CD44, respectively. Our study implicates miR-302a as a candidate prognostic predictor and a therapeutic agent in CRC.

## Introduction

Colorectal cancer (CRC) is the third leading cause of new cancer cases and the second most frequent cause of cancer-related death 1. Increasing frequencies of metastasis and drug resistance contribute to the poor prognosis of CRC patients. An estimated 50%-60% of CRC patients may have metastases; the 5-year overall survival rate of metastatic CRC (mCRC) patients is only 13.3%, while that of patients in the localized stage is 91.1% 2. Cetuximab (CTX), a monoclonal antibody targeting EGFR, is recommended as a first-line therapy for mCRC in the National Comprehensive Cancer Network (NCCN) guidelines 3. However, approximately 70% of patients without primary resistance still progress within 3 to 12 months after starting CTX therapy 4.

Increasing evidence suggests that the triggering of metastasis and the development of drug resistance in cancer cells have certain connections 5. Previous studies have reported that drug resistance is often accompanied by a higher metastatic ability in resistant cells than in their parental sensitive cells 5. More recently, several studies have revealed that the same genes could simultaneously regulate the development of drug resistance and metastasis. For instance, loss of the tumor suppressor FAT1 promotes resistance to CDK4/6 inhibitors and increases migration and invasion 6, 7. The overexpression of P-gp, an efflux membrane pump that transfers cytotoxic agents, triggers resistance to cytotoxic drugs and metastasis 8. Depletion of the E3 ubiquitin ligase HectD1 increases metastasis and confers resistance to the cytotoxic drug cisplatin 9. All these reports suggest that coordinately regulatory mechanisms exist during cancer cells developing drug-resistant and metastatic characteristics. However, whether epigenetic regulation plays a role in these processes remains unclear.

microRNAs (miRNAs) are single-stranded noncoding RNAs that contain 22-24 nucleotides and post-transcriptionally regulate gene expression by blocking mRNA translation or degrading target mRNAs. Importantly, one miRNA usually targets multiple mRNAs through incomplete complementary base pairing, suggesting that miRNAs can regulate a wide variety of cellular processes 10. We previously established a pair of CTX-sensitive and CTX-resistant CRC cell lines based on a three-dimensional (3D) cell culture system, and multiple miRNAs were determined to be differentially expressed by small RNA-Seq 11. Among these miRNAs, miR-302a was significantly downregulated in the CTX-resistant cells, suggesting that its reduction may be involved in the acquisition of CTX resistance. Interestingly, previous studies have reported that miR-302a overexpression inhibits cell migration and invasion in several cancer types 12-14, supporting the hypothesis that miR-302a may simultaneously regulate drug resistance and metastasis in CRC. However, the underlying mechanism is largely unknown. Moreover, the function of miR-302a in CRC remains ambiguous according to previous reports. Although some studies have shown that miR-302a may function as a tumor suppressor by using CRC cells* in vitro* 14, 15, another report found that miR-302a expression was higher in CRC tissues than in normal colonic mucosa 16. These seemingly contradictory reports urge further investigation into miR-302a expression and function in CRC.

In this study, we examined miR-302a expression in a panel of CRC cell lines, patient-derived xenografts (PDXs), and large-scale tissue microarrays. We identified that miR-302a expression was decreased in most CRC cells and PDXs and was often reduced in CRC tissues. The downregulation of miR-302a correlated with the poor prognosis of CRC patients. miR-302a overexpression suppressed CRC cell metastasis and restored CTX responsiveness both *in vitro* and *in vivo*. Mechanistically, miR-302a inhibited metastasis by directly targeting nuclear factor I B (NFIB), which was identified as a transcriptional activator of integrin subunit alpha 6 (ITGA6). miR-302a restored CTX responsiveness by directly suppressing CD44, which induced cancer stem cell (CSC)-like properties and activated EGFR-dependent MAPK and AKT signaling. These results identify important roles for miR-302a in regulating metastasis and CTX resistance with predictive and therapeutic implications in CRC.

## Materials and methods

### Cell culture

The human CRC cell lines HCT116, DiFi, HCT8, HT29, LoVo, RKO, DLD-1, SW620, SW480, Caco-2, SW1463, T84, and SW948, the normal human intestinal epithelial cell line HIEC, CTX-sensitive CC cells and CTX-resistant CC-CR cells have been previously described 11 and were cultured in DMEM (Gibco) with 10% fetal calf serum (Gibco), 1% antibiotic-antimycotic (Gibco), 1% L-glutamine and 1% MEM nonessential amino acids (Gibco) at 37°C in 5% CO_2_. The complete medium used for culturing CC-CR cells contained 3 µg/ml CTX (Merck). The cell lines were recently authenticated by cellular morphology and short tandem repeat analysis using the AmpF/STR Identifier Kit (Applied Biosystems). All cells were tested for mycoplasma every 3 months and were negative. Cells with negative detection results were passaged 3 days after thawing and then frozen for long-term storage. All experiments involving the cell lines were performed within five passages. The 3D culture protocol has been previously described 11. Colonies of CC and CC-CR cells were lysed for RNA extraction after 14 days or fixed in 4% paraformaldehyde (PFA) for counting (Oxford Optronix GelCount) after 21 days. Origin and genetic information of cell lines used in the study is listed in Table S1.

### Human CRC tissue samples

Fresh paired human CRC tissues and the corresponding adjacent nontumorous colorectal tissues were collected after receiving informed consent from 20 patients who were treated with surgical resection alone between 2014 and 2015 at the Xijing Hospital of Digestive Diseases (Xi'an, China). All samples were clinically and pathologically diagnosed. This study was approved by Xijing Hospital's Protection of Human Subjects Committee. Informed consent was obtained from each patient. All tissue samples for RNA and protein extraction were snap-frozen in liquid nitrogen within 1 h of surgical resection and stored in liquid nitrogen until analyzed. Archived formalin-fixed paraffin-embedded (FFPE) tissue samples for fluorescence in situ hybridization (FISH) and immunochemical (IHC) staining from the same patients were collected. Tissue microarrays (TMA 1 and 2) were obtained from Shanghai Outdo Biotech Co., Ltd. The clinicopathological information of the CRC patients included in this study is shown in Table S2.

### Establishment of patient-derived xenografts (PDXs)

Fresh tumor tissues were obtained from CRC patients who underwent surgical resection at the Xijing Hospital of Digestive Diseases. All those CRC patients harbored wild-type *KRAS*/*BRAF* genes. The tumor tissues were collected and sliced into 1-3 mm^3^ fragments and subcutaneously implanted into 6- to 8-week-old NSG nude mice. After implantation, the condition of the mice and tumor growth were monitored twice per week. The tumor volume was assessed by bilateral caliper measurements and calculated using the formula: tumor maximum diameter (L) × the right angle diameter to that axis (W)^2^/2. Once the xenograft reached 100 mm^3^, mice were then randomly categorized into two groups and treated with either 1 mg/animal CTX or 0.9% saline intraperitoneally twice a week for three weeks. CTX responsiveness was assessed based on the ratio of the average volume in the treatment group to that in the control group. A classification of “CTX highly responsive” (ratio<0.1), “CTX responsive” (ratio between 0.1 and 0.42), or “CTX resistant” (ratio>0.42) was assigned to each mouse in the CTX-treated group. RNA was extracted from xenografts and miR-302a expression was detected in CTX resistant group and control group. All animal studies were conducted according to the Association for the Assessment and Accreditation of Laboratory Animal Care and the Institutional Animal Care and Use Committee guidelines.

### Constructs, oligonucleotides, infection and transfection

The miR-302a mimic, inhibitor and corresponding control were synthesized by RiboBio (Guangzhou, China). Chemically modified small interfering RNAs (siRNAs) for NFIB, CD44, and ITGA6 were synthesized by GenePharma (Shanghai, China). The constructs containing the pre-miR-302a or NFIB, CD44, ITGA6 shRNA sequences were cloned into the lentiviral GV369 vector (GeneChem). Expression vectors encoding NFIB and CD44 were constructed into the GV358 vector (GeneChem) between the AgeI and AgeI sites for expression driven by the CMV promoter. A luciferase expression vector (LV11 vector) that contains the CMV promoter was purchased from GenePharma. An empty lentiviral vector was used as a negative control. The 3'-UTR fragments of NFIB or CD44 containing putative miR-302a target sites were amplified and cloned downstream of the SV40 promoter-driven *Renilla* luciferase cassette between the XhoI and NotI sites in the psiCHECK-2 vector (Promega).

To generate stable cell lines, the indicated cells were infected with lentiviruses at a multiplicity of infection of 20:1 in a solution containing 5 µg/ml polybrene and selected with 2 µg/ml puromycin for 1 week. The indicated cells were transfected with miR-302a mimic (RiboBio) or nontargeting negative control oligos using Lipofectamine RNAiMax (Life Technologies) according to the manufacturer's instructions. After 48 h, the infected or transfected cells were harvested for *in vitro* functional experiments or RNA isolation. After 72 h, the infected or transfected cells were harvested for protein extraction. All shRNA information is listed in Table S3.

### RNA extraction and qPCR

Total RNA was extracted from cultured cells and surgically resected fresh-frozen CRC tissue using the miRNeasy kit (QIAGEN) according to the manufacturer's instructions. RNA quality was examined by A260/A280 absorption. The complementary DNA (cDNA) to be used for detecting miRNAs was synthesized with a TaqMan miRNA reverse transcription kit (Takara). For mRNA detection, 500 ng of total RNA was used for cDNA synthesis in a 10-µl system with a PrimeScript^TM^ RT Master Mix kit (Takara). qPCR was performed in triplicate using SYBR^®^ Premix Ex Taq^TM^ II (Takara) with the CFX96™ QPCR Detection System (Bio-Rad). The primers specific to mature miR-302a were purchased from RiboBio (Guangzhou, China). The primers for the other genes of interest were synthesized by Takara. U6 and GAPDH were used as internal controls. The 2^-ΔΔCT^ method was used to determine the fold changes in the mRNA levels between each sample and the reference sample. Primer information is listed in Table S4.

### Protein extraction and western blot

Cultured cells and CRC tissue were lysed in RIPA Lysis Buffer (Beyotime) supplemented with a protease inhibitor and phosphatase inhibitor (Roche). Protein lysates were separated by SDS-PAGE and transferred onto nitrocellulose membranes (Millipore). The membranes were sequentially blocked with 10% nonfat milk, incubated with primary antibodies, and incubated with secondary HRP-conjugated antibodies (Cell Signaling Technology). The primary antibody information is listed in Table S5. The immunoreactive protein complexes were detected with enhanced chemiluminescence reagents (ZETA) and scanned by the Molecular Imager ChemiDox XRS+ Imaging System with Image Lab software (Bio-Rad).

### Migration, invasion and wound-healing assays

*In vitro* cell migration (Millipore) and invasion (Corning) assays were conducted using chambers (24-well) with 8-mm-diameter micropore membranes without Matrigel (for the migration assay) or with Matrigel (for the invasion assay) according to the manufacturer's instructions. Briefly, cells that were resuspended using serum-free medium were seeded into each upper compartment of the chamber, with the bottom compartment medium containing 20% fetal calf serum. After 16-60 h of incubation at 37°C with 5% CO_2_, the migratory cells were fixed with ethyl alcohol and stained with 0.1% crystal violet. Three fields were randomly chosen for quantification. A wound-healing assay was performed using a Culture-Insert 2 well (ibidi), which contains two wells separated by a 500-µm-thick wall. Cells were assessed in three random fields with the healing wound centered in the field at 0 h and 48 h after removing the wall to quantify cell migration.

### Tumor sphere formation assay

A total of 1.5×10^4^ cells/well were cultured in serum-free DMEM/F12 (Gibco) supplemented with 1% antibiotic-antimycotic (Gibco), 10 ng/ml epidermal growth factor (PeproTech), and 10 ng/ml basic fibroblast growth factor (PeproTech) in ultralow-attachment 6-well plates (Corning). The spheres were captured and counted when the sphere diameter reached 50-250 µm (approximately 1-2 weeks). Spheres with a diameter of 50-250 µm were counted.

### Cell Counting Kit 8 (CCK-8) assay

Caco-2 and DiFi cells with a cell density of 2×10^3^ cells/well were seeded in 96-well plates. After incubating for 24 h, the cells were treated with varying concentrations of CTX and incubated for 72 h at 37°C in 5% CO_2_, and then the cells were incubated with CCK-8 solution (Dojindo, Kumanoto, Japan) for 2.5 h. A Varioskan^®^ Flash spectrometer (Thermo Fisher Scientific, Waltham, USA) was used to measure the absorbance at 450 nm.

### LIVE/DEAD cell viability assay

Caco-2 cells with a cell density of 5×10^4^ were seeded in 12-well plates. After incubating for 24 h, the cells were treated with 100 µg/ml CTX and incubated for 72 h at 37°C in 5% CO_2_. The cells were then washed with culture-grade DPBS to eliminate or reduce serum esterase activity and stained with buffer containing 2 μM calcein AM and 4 μM ethidium homodimer-1 (Thermo Fisher Scientific). The cells were stained for 30 min at room temperature. Live cells were visualized as green with excitation at 494 nm and emission at 517 nm, and dead cell nuclei were visualized as red with excitation at 517 nm and emission at 617 nm. The number of dead cells and the total cell count were computed, and the survival rate was calculated for each field/image. Each assay was performed in triplicate.

### *In vivo* metastasis assay

Female nude mice aged 6-8 weeks were provided by the Experimental Animal Center of the Fourth Military Medical University and housed in pathogen-free conditions. Animal studies were approved by the Fourth Military Medical University Animal Care Committee. Stable miR-302a-overexpressing SW620 cells labeled with luciferase were harvested by trypsinization, washed and resuspended in PBS. A total of 5×10^6^ cells in 200 µl of PBS were injected via the tail vein into the nude mice. Six weeks after injection, the bioluminescence intensity was evaluated using the IVIS Spectrum *in vivo* imaging system (PerkinElmer, Shanghai, China). After the mice were euthanized, the lung tissues were fixed in 4% PFA, and hematoxylin and eosin (H&E) staining was performed to evaluate the number of metastases.

### *In vivo* tumor growth in a xenograft model

The acquisition and housing of the nude mice used as xenograft models matched the conditions described for the *in vivo* metastasis assays. Animal studies were approved by the Fourth Military Medical University Animal Care Committee. Stable miR-302a-overexpressing Caco-2 cells labeled with luciferase were harvested by trypsinization, washed with PBS, and resuspended in PBS. A total of 5×10^6^ cells in 150 µl of PBS were subcutaneously injected into the flanks (the left flank was injected with miR ctrl cells, and the right flank was injected with miR-302a cells). When the tumor size reached approximately 100 mm^3^ (approximately 2 weeks after injection), the mice were randomly assigned to the control and CTX treatment groups. CTX was injected intraperitoneally at a dose of 0.3 mg/mouse twice weekly. The tumor size was measured every six days. One month after the CTX treatment, the mice were sacrificed according to institutional ethical guidelines, and the tumor size and weight were measured. Subsequently, the tumors were paraffin-embedded for H&E staining and IHC staining for NFIB or CD44. Additionally, Ki-67 and Cleaved Caspase-3 staining were performed to further evaluate tumor growth and the efficacy of CTX treatment.

### Dual-luciferase reporter assay

Caco-2 cells were seeded in a 24-well plate and cotransfected with 0.5 µg of dual-luciferase 3'-UTR vector and 50 nM miR-302a mimic or 100 nM miR-302a inhibitor using Lipofectamine 2000 (Invitrogen). The cells were harvested 48 h after transfection, and the firefly and *Renilla* luciferase activities were examined using the Dual-Luciferase Reporter Assay System (Promega).

### Chromatin immunoprecipitation (ChIP)

The Pierce Agarose ChIP Kit (Thermo Fisher Scientific) was used for the ChIP assays, and ChIP-enriched DNA samples were analyzed by PCR. Cells were cross-linked with 1% formaldehyde for 10 min at 20°C and quenched in glycine. The primary antibody information is listed in Table S5. The DNA from the cells was recovered and subjected to PCR to amplify the binding sites in the ITGA6 promoter region. PCR was performed and visualized by gel electrophoresis. The PCR primer sets are listed in Table S4.

### Immunofluorescence (IF)

SW1463 cells were plated onto glass coverslips (Millipore), fixed with 4% PFA for 10 min, and permeabilized with 0.1% Triton X-100 in PBS for 15 min. Blocking solution was applied for 30 min at room temperature. The primary antibodies were applied at 4°C overnight. The primary antibody information is listed in Table S5. The secondary antibody was Alexa Fluor 568-conjugated or 488-conjugated goat anti-rabbit IgG antibody (Life Technologies) and was applied for 1 h at room temperature. Nuclei were stained with Hoechst 33342. IF staining of transfected CC-CR cells in 3D culture was performed as previously described 17. Slides were mounted with Mounting Medium (Vectashield) before imaging with a Nikon ECLIPSE Ti confocal microscope.

### Fluorescence* in situ* hybridization (FISH)

FISH assays were performed to detect miR-302a in CRC tissue as previously described 18. miR-302a probes, including a double digoxigenin (DIG)-labeled probe against miR-302a, were synthesized (Exiqon). An anti-digoxigenin HRP-conjugated antibody (PerkinElmer) and tyramide signal amplification (TSA) Cy3 (PerkinElmer) were used. Nuclei were stained with Hoechst 33342. Slides were mounted with Mounting Medium (Vectashield) before imaging with a Nikon ECLIPSE Ti confocal microscope. The staining intensity was assessed as previously described 19.

### Immunohistochemical (IHC) staining

IHC staining for the target molecules was performed on tissue microarrays, single sections made from clinical FFPE tissues and xenograft tumor samples. The samples were deparaffinized and subjected to heat-mediated antigen retrieval with Tris/EDTA buffer pH 9.0 (ITGA6) or 10 mM sodium citrate buffer pH 6.0 (NFIB, CD44, Ki-67 and Cleaved Caspase-3) according to the manufacturer's instructions. The slides were treated with 3% H_2_O_2_ and then incubated overnight at 4°C with a primary antibody. The primary antibody information is listed in Table S5. The slides were then incubated with secondary antibodies and visualized using 3,3-diaminobenzidine tetrahydrochloride plus (DAB+). Counterstaining for nuclei was performed using hematoxylin. The staining intensity was assessed as previously described 20.

### Statistical analysis

Each experiment was repeated two or three times or more as described in each figure legend. IBM SPSS version 23 software was used for the statistical analysis. The data are represented as the mean ± SEM. Statistical significance was determined using a two-tailed paired or unpaired Student's *t* test, χ^2^ test, or nonparametric signed-rank test according to the type of experiment and homogeneity of the variance. The correlation between the expression of miR-302a and its target genes was determined by Spearman correlation analysis. Survival curves were analyzed by the Kaplan-Meier method, and a log-rank test was used to assess significance. Statistical significance is indicated by *P* values less than 0.05.

### Data availability

The authors declare that all data supporting the findings of this study are available within the article and its Supplementary Information Files or from the corresponding author upon reasonable request.

## Results

### miR-302a expression is downregulated in CRC

**Figure 1:**
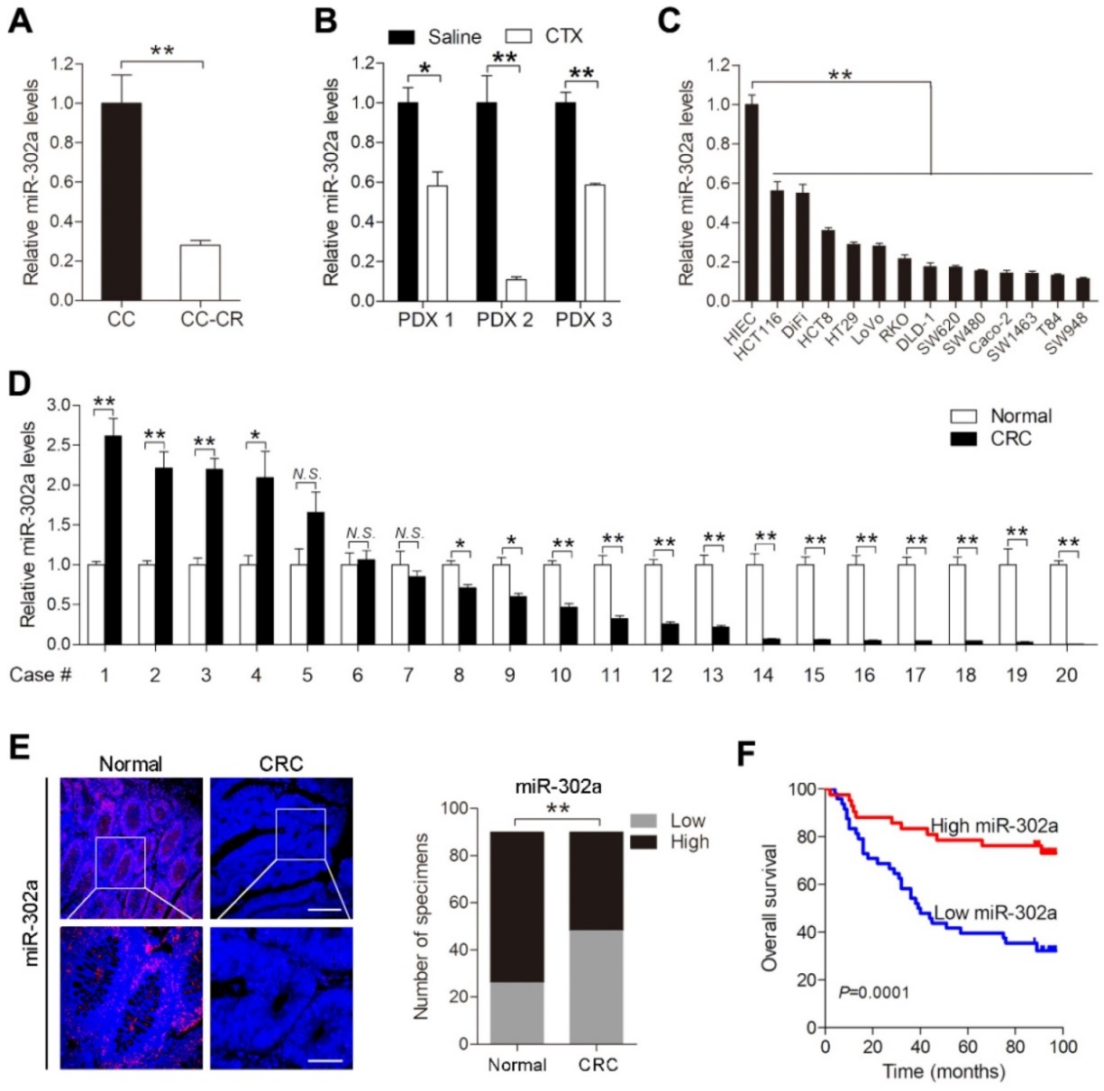
** miR-302a expression is downregulated in CRC. A, B** qPCR analysis was used to assess miR-302a expression in cetuximab (CTX)-resistant (CC-CR) and CTX-sensitive (CC) cell lines in a 3D culture system (A) and in 3 CTX-resistant patient-derived xenografts (PDXs) (B). **C, D** qPCR analysis was used to assess miR-302a expression in 13 human CRC cell lines, an immortalized normal human intestinal epithelial cell line, HIEC (C), and 20 paired CRC tissue and adjacent normal tissue samples (D). **E** Representative images and analysis of fluorescence *in situ* hybridization (FISH) assays for miR-302a in 90 paired CRC and adjacent normal tissue samples are shown. Scale bar, 100 μm (top) and 50 μm (bottom). **P<0.01, χ^2^ test. **F** Kaplan-Meier overall survival curves were constructed for CRC patients (n=90) who were divided into low- and high-miR-302a expression groups. Significance was determined by log-rank test. Transcript levels were normalized to U6 expression. The values represent the mean ± SEM. A-D *P<0.05, **P<0.01, Student's *t* test. *N.S.*, not significant. Data are representative of three independent experiments.

We previously established a pair of CTX-resistant (CC-CR) and CTX-sensitive (CC) cell lines in a 3D culture system. Small RNA-Seq revealed that miR-302a expression was significantly decreased in CC-CR cells compared with CC cells 11. qPCR validated the downregulation of miR-302a in CC-CR cells (Figure 1A). We further detected miR-302a expression in PDXs and found that its expression was decreased in CTX-resistant xenografts compared with saline-treated xenografts (Figure 1B). Next, we examined miR-302a expression in a panel of CRC cell lines and found that miR-302a was decreased in 13 lines compared with the immortalized human normal intestinal epithelial cell line HIEC (Figure 1C). miR-302a expression was also decreased in 14/20 (70%) CRC tissues compared with the paired adjacent normal tissues (Figure 1D). Furthermore, FISH assays were conducted using a tissue microarray (TMA 1) including 90 paired CRC tissues and adjacent normal tissues, and miR-302a expression was found to be decreased in most CRC tissues (Figure 1E). Kaplan-Meier analysis showed that CRC patients with lower miR-302a expression exhibited shorter overall survival than CRC patients with higher miR-302a expression (Figure 1F). Correlation analysis revealed that lower miR-302a expression was positively correlated with CRC tumor size, histological grade differentiation, and tumor stage (Table S6).

### miR-302a inhibits CRC cell migration and invasion and restores CTX responsiveness *in vitro*

**Figure 2:**
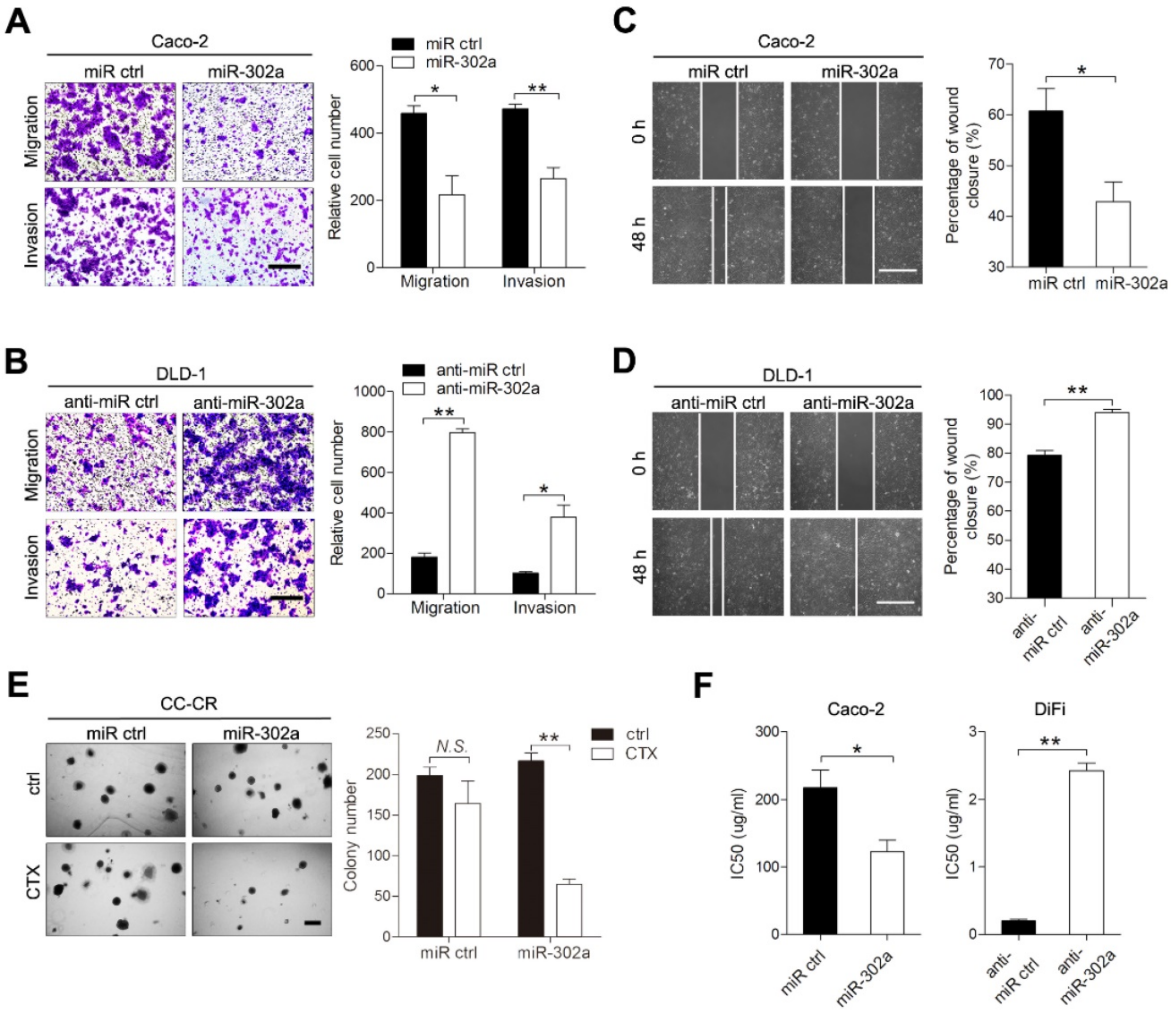
** miR-302a inhibits migration and invasion and restores CTX responsiveness in CRC cells. A, B** Representative images and graph of up- and downregulation of miR-302a in Caco-2 (A) and DLD-1 (B) cells, as determined by Transwell migration and invasion assays. Scale bar, 500 μm. **C, D** Representative images and graph of miR-302a up- and downregulation in Caco-2 (C) and DLD-1 (D) cells, as determined by wound-healing assay. Scale bar, 500 μm. **E** The colony number of miR-302a-overexpressing CC-CR cells treated with CTX in a 3D culture system. Scale bar, 200 μm.** F** The IC50 of CTX with miR-302a up- or downregulation in Caco-2 or DiFi cells. The values represent the mean ± SEM. *P<0.05, **P<0.01, Student's *t* test.* N.S.*, not significant.

To investigate the role of miR-302a in CRC, we established gain- and loss-of-function cell models by infecting CRC cells with lentivirus that carried miR-302a or targeted miR-302a (anti-miR-302a) using DLD-1 and Caco-2 cells, in which miR-302a is moderately expressed. Upon miR-302a overexpression, the migratory and invasive abilities of Caco-2 and DLD-1 cells were markedly decreased (Figure 2A and Figure S1A). Conversely, miR-302a downregulation enhanced migration and invasion in DLD-1 and Caco-2 cells (Figure 2B and Figure S1B). Wound-healing assays also indicated that miR-302a could inhibit CRC cell migration (Figure 2C-D, Figure S1C-D). We next examined whether miR-302a could regulate CTX resistance in Caco-2 and DiFi cells, which have wild-type *KRAS* and *BRAF* genes and are suitable for CTX treatment. The colony number of CC-CR cells cultured in 3D significantly decreased upon CTX treatment when miR-302a was overexpressed (Figure 2E). In addition, miR-302a upregulation in Caco-2 cells (partially responsive to CTX) decreased the 50% inhibitory concentration (IC50) of CTX, and miR-302a downregulation in DiFi cells (extremely sensitive to CTX) increased the IC50 of CTX, as measured by CCK-8 assay (Figure 2F). In addition, we performed LIVE/DEAD cell viability assays using Caco-2 cells that stably expressed miR-302a or controls to detect cell death after CTX treatment. Cell death increased after CTX treatment and this effect further enhanced when miR-302a was overexpressed (Figure S1E). These results indicate that miR-302a inhibits migration and invasion and restores CTX responsiveness in CRC cells.

### miR-302a inhibits CRC metastasis and restores CTX responsiveness *in vivo*

**Figure 3:**
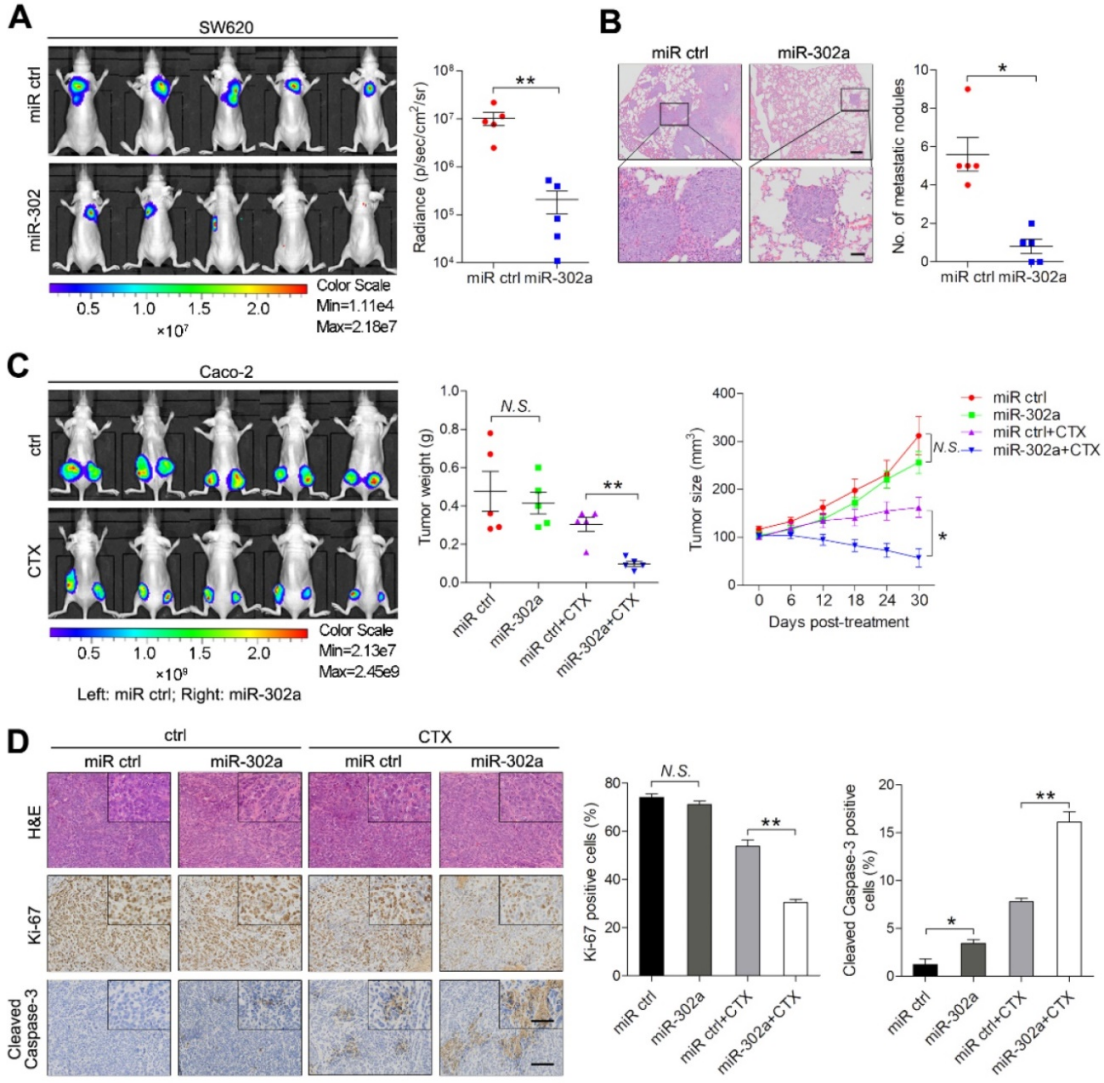
** miR-302a inhibits CRC metastasis and restores CTX responsiveness *in vivo*. A** Bioluminescent images and radiance values of mice injected with control or miR-302a-overexpressing SW620 cells (n=5). **P<0.01, Mann-Whitney U test. **B** Representative H&E staining images and graph of metastatic nodule numbers in the lung tissue sections from nude mice injected with SW620 cells stably expressing miR-302a or miR ctrl (n=5). *P<0.05, Mann-Whitney U test. Scale bar, 200 μm (top) and 50 μm (bottom). **C** Bioluminescent images (left) and graph of tumor weights (middle; **P<0.01, paired Student's *t* test) and volumes (right; *P<0.05, repeated-measures ANOVA) of xenografts treated with CTX from mice implanted with control or miR-302a-overexpressing Caco-2 cells (n=5). **D** Representative images of H&E, Ki-67 and Cleaved Caspase-3 staining and quantification of the positive staining of cells in xenografts from nude mice implanted with control or miR-302a-overexpressing Caco-2 cells. *P<0.05, **P<0.01, Student's *t* test. Scale bar, 50 μm (insert) and 100 μm (main). The values represent the mean ± SEM. *N.S.*, not significant.

To examine the effect of miR-302a on metastasis, highly metastatic CRC cell line SW620 stably expressing miR-302a or a negative control (miR ctrl) were delivered into nude mice by tail vein injection. After 6 weeks, *in vivo* bioluminescence imaging showed that the luminescent intensities in the lungs were substantially lower in mice injected with SW620 cells stably expressing miR-302a than in mice in the control group (Figure 3A). Consistently, the number of metastatic nodules in the lungs was decreased after miR-302a overexpression (Figure 3B). These results indicate that the ectopic expression of miR-302a inhibited CRC metastasis *in vivo*. To determine whether miR-302a affects CTX resistance *in vivo*, Caco-2 cells stably expressing miR-302a or miR ctrl were injected subcutaneously into nude mice. CTX was injected intraperitoneally after the tumor volume reached approximately 100 mm^3^. The tumor size and weight were significantly reduced by CTX treatment in the mice implanted with miR-302a-overexpressing Caco-2 cells (Figure 3C and Figure S1F). IHC staining showed that xenografts from the miR-302a-overexpression group presented less Ki-67 staining and more Cleaved Caspase-3 staining than those from the control group upon CTX treatment (Figure 3D). These results indicate that the induction of miR-302a expression inhibits metastasis and restores CTX responsiveness *in vivo*.

### NFIB and CD44 are direct targets of miR-302a

**Figure 4:**
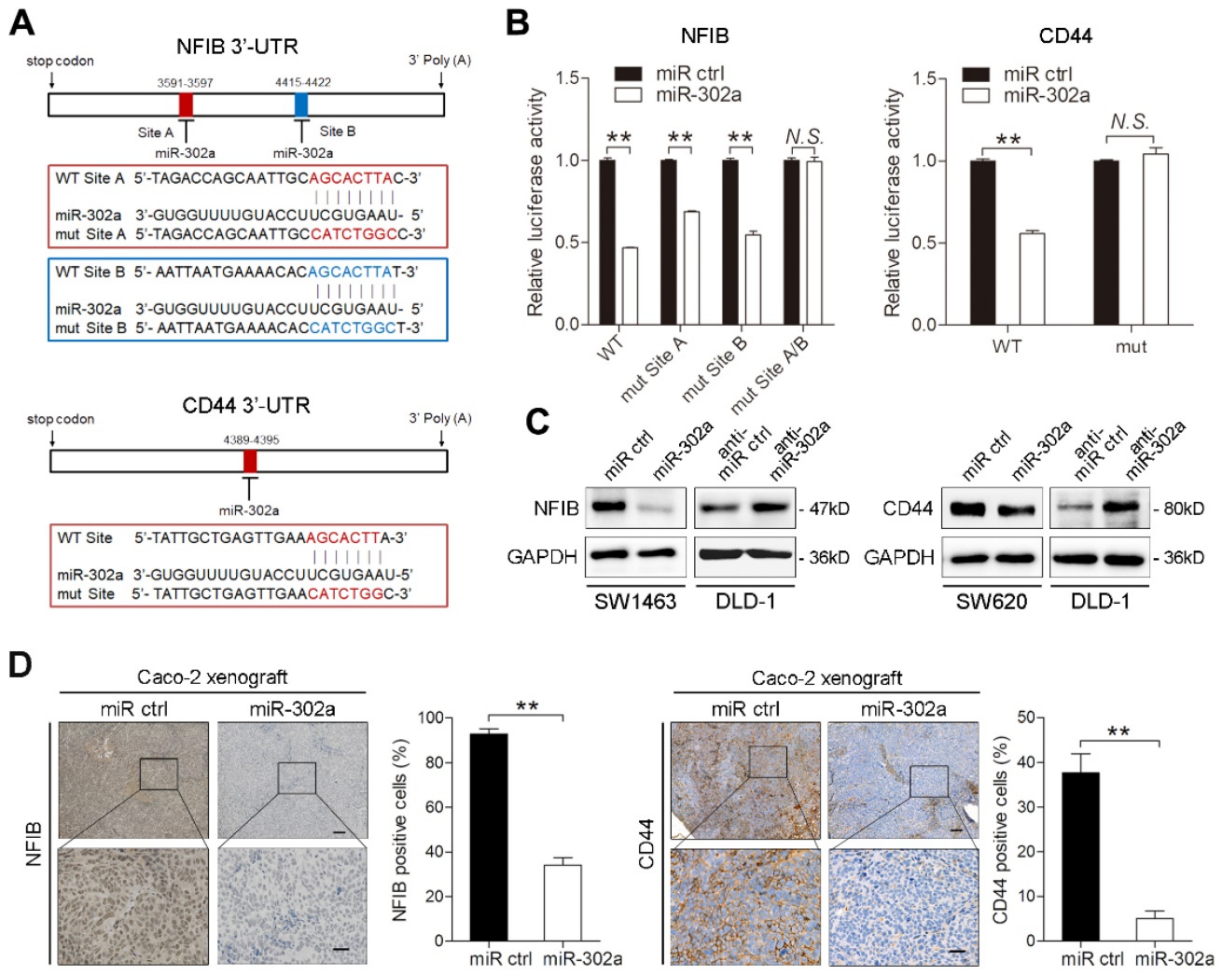
** miR-302a directly targets NFIB and CD44 in CRC cells. A** Diagram of 3'-UTR of NFIB (top) or CD44 (bottom) 3'-UTR-containing reporter construct. Mutations were generated at the predicted miR-302a binding sites located in the indicated 3'-UTR. **B** Luciferase reporter assay performed with Caco-2 cells transfected with miR-302a mimics or control oligomers and NFIB or CD44 3'-UTR wild-type or mutant binding site reporter. **C** Western blot analysis of NFIB and CD44 expression in the indicated cells. **D** Representative NFIB (left) and CD44 (right) staining images and quantification of the positive staining of cells in the xenografts from nude mice implanted with control or miR-302a-overexpressing Caco-2 cells. Scale bar, 100 μm (top) and 20 μm (bottom). The values represent the mean ± SEM. **P<0.01, Student's *t* test. *N.S.*, not significant.

To identify putative targets of miR-302a in CRC, we conducted bioinformatic analyses using 5 miRNA target prediction algorithms, including TargetScan, PicTar, RNA22, PITA and miRanda. Among candidate miR-302a targets (Table S7), NFIB and CD44 were both identified to harbor the binding site of miR-302a in their 3'-UTR in 4 databases (Figure 4A). NFIB is a transcription factor that has been implicated in driving a highly migratory phenotype by enhancing cell-cell adhesion and motility 21. CD44 is a transmembrane glycoprotein involved in cancer proliferation, metastasis, and drug resistance 22. Thus, we focused on NFIB and CD44 in further investigations. To verify whether miR-302a directly binds to the 3'-UTRs of NFIB and CD44, we performed dual-luciferase reporter assays in Caco-2 cells. miR-302a overexpression suppressed the luciferase activities of both the NFIB and CD44 3′-UTR reporter constructs, whereas this effect was abolished when mutations were introduced into the miR-302a binding sequences in these constructs (Figure 4B). NFIB and CD44 protein expression decreased after miR-302a overexpression, while miR-302a knockdown increased NFIB and CD44 protein levels (Figure 4C and Figure S1G). The inhibitory effect of miR-302a on NFIB and CD44 was dose-dependent (Figure S1H). Moreover, NFIB and CD44 staining was also decreased in the Caco-2 xenografts stably expressing miR-302a compared with the control xenografts (Figure 4D). These results indicate that miR-302a directly targets NFIB and CD44 in CRC cells.

### miR-302a inhibits metastasis by suppressing NFIB-mediated ITGA6 transcription

**Figure 5:**
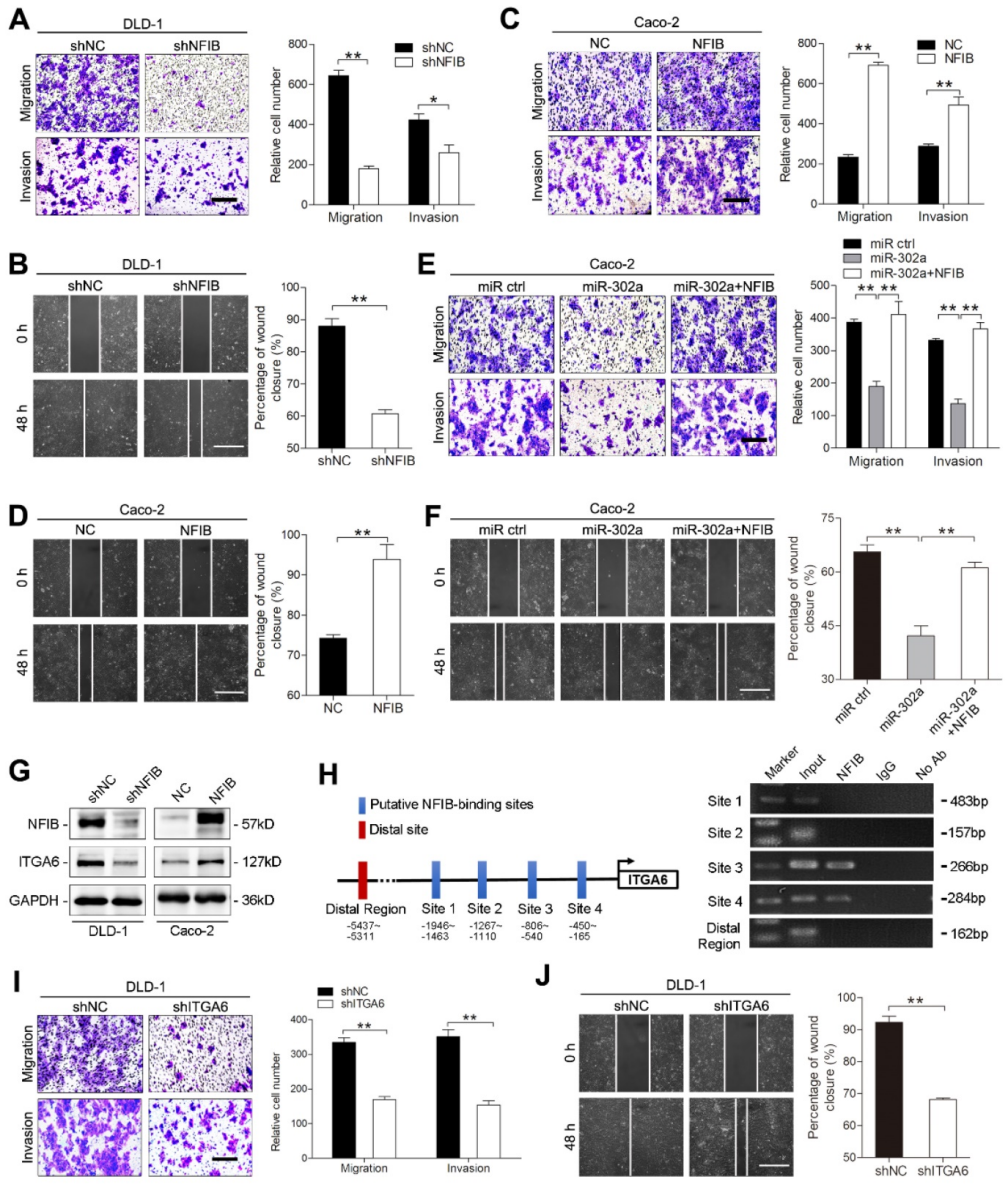
** miR-302a inhibits NFIB/ITGA6 axis-mediated migration and invasion in CRC cells. A, B** Representative images and graph of NFIB downregulation in DLD-1 cells, as determined by Transwell migration and invasion assays (A) and wound-healing assay (B). Scale bar, 500 μm. **C, D** Representative images and graph of NFIB down- or upregulation in Caco-2, as determined by Transwell migration and invasion assays (C) and wound-healing assay (D). Scale bar, 500 μm.** E, F** Representative images and graph of miR-302a overexpression in combination with or without NFIB upregulation, as determined by Transwell migration and invasion assays (E) and wound-healing assay (F). Scale bar, 500 μm.** G** Western blot analyses of ITGA6 expression with NFIB up- or downregulation in the indicated cells. **H** Schematic presentation of NFIB binding sites in the ITGA6 promoter region (left) and gel electrophoresis image of the ChIP analysis (right). **I, J** Representative images and graph of ITGA6 downregulation in DLD-1 cells, as determined by Transwell migration and invasion assays (I) and wound-healing assay (J). Scale bar, 500 μm. The values represent the mean ± SEM. *P<0.05, **P<0.01, Student's *t* test.

Several studies have reported the involvement of NFIB in metastasis 21, 23, 24. We questioned whether miR-302a inhibits CRC metastasis by suppressing NFIB. To this end, we overexpressed or silenced NFIB with lentiviral constructs in DLD-1 and Caco-2 cells and verified the changes in NFIB expression (Figure S2A-B). NFIB knockdown reduced the migration and invasion of DLD-1 and Caco-2 cells (Figure 5A-B and Figure S2C-D). Conversely, NFIB overexpression enhanced cell migration and invasion (Figure 5C-D and Figure S2E-F). Furthermore, the transduction of NFIB into the miR-302a-overexpressing Caco-2 cells abrogated the inhibitory effect of miR-302a on migration and invasion (Figure 5E-F). These results indicate that NFIB is a functional target of miR-302a in CRC cells.

Since NFIB belongs to a transcription factor family, we asked whether the metastasis-promoting effect of NFIB is mediated by its transcriptional effectors. We found that ITGA6, which interacts with the extracellular matrix and drives metastasis in multiple cancers 25-27, was a potential target of NFIB according to a published ChIP-Seq dataset from the GEO repository 21 and identified multiple NFIB binding sites in the promoter region of ITGA6 (Figure 5H). NFIB knockdown decreased ITGA6 expression, and NFIB overexpression increased ITGA6 levels in DLD-1 and Caco-2 cells (Figure 5G and Figure S2G). ChIP assays confirmed the direct binding of NFIB to the ITGA6 promoter region located from -806 to -165 bp of its transcription start site (Figure 5H). As expected, the suppression of ITGA6 significantly decreased the migration and invasion of DLD-1 and Caco-2 cells (Figure 5I-J, Figure S2H-J). Together, these results suggest that miR-302a regulates CRC metastasis through NFIB-mediated ITGA6 transcription.

### miR-302a sensitizes CRC to CTX by suppressing CD44-mediated CSC-like properties and EGFR-dependent signaling

**Figure 6:**
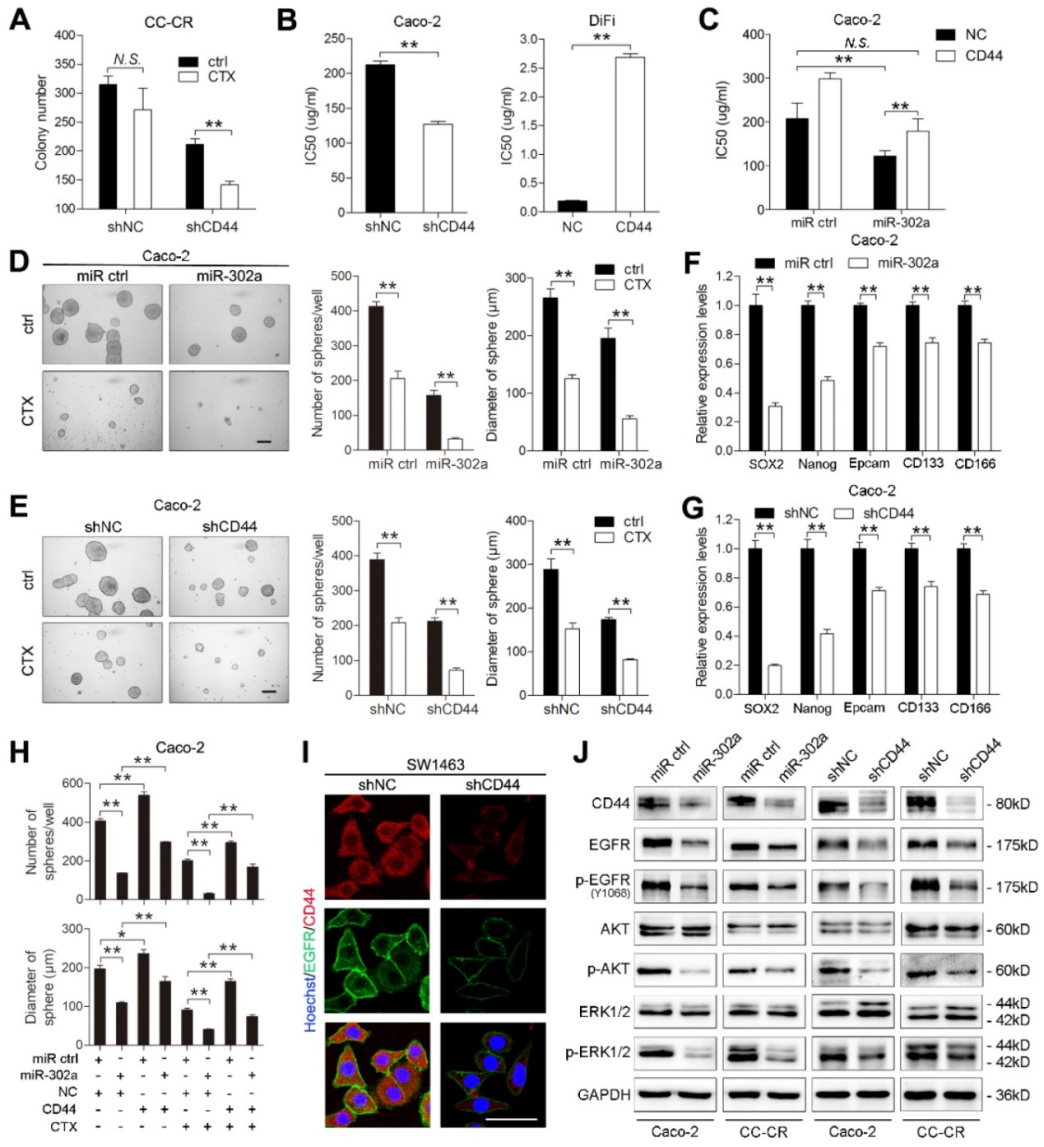
** miR-302a restores CTX responsiveness by suppressing CD44 in CRC cells. A** Colony number of CC-CR cells when transfected with shCD44 or control vector in 3D culture system in the presence or absence of CTX (10 μg/ml).** B** IC50 of CTX with CD44 up- or downregulation in Caco-2 or DiFi cells, as determined by CCK-8 assay. **C** IC50 of CTX with miR-302a overexpression combination with or without CD44 upregulation in Caco-2 cells, as determined by CCK-8 assay. **D, E** Representative micrographs (left) and the number (middle) and diameter (right) of tumor spheres formed with miR-302a upregulation (D) and CD44 downregulation (E) in Caco-2 cells in the presence or absence of CTX (100 μg/ml). Scale bar, 200 μm. **F, G** qPCR analysis of SOX-2, Nanog, Epcam, CD133 and CD166 expression in Caco-2 cells with miR-302a upregulation (F) and CD44 downregulation (G). GAPDH was used as a negative control. **H** Quantification of the number (top) and diameter (bottom) of tumor spheres formed when transfected with miR-302a and/or CD44 in Caco-2 cells in the presence or absence of CTX (100 μg/ml). **I** Representative immunofluorescence images of CD44 and EGFR staining in SW1463 cells with CD44 downregulation. Scale bar, 50 μm.** J** Western blot analysis of CD44, EGFR, p-EGFR (Y1068), AKT, p-AKT, ERK1/2 and p-ERK1/2 expression in the indicated CRC cells. The values represent the mean ± SEM. *P<0.05, **P<0.01, Student's *t* test. *N.S*., not significant.

CD44 has been reported to promote cancer cells developing resistance to cancer therapies 28, 29. We thus speculated that miR-302a restored CTX responsiveness in CRC cells by targeting CD44. CD44 knockdown modestly reduced the colony number of CC-CR cells in 3D culture, whereas the reduction in colony number was more pronounced in the presence of CTX (Figure 6A). We then overexpressed and silenced CD44 with lentiviral constructs in DLD-1 and Caco-2 cells (Figure S3A-B). The IC50 of CTX decreased when CD44 was knocked down in Caco-2 cells and increased when CD44 was overexpressed in DiFi cells (Figure 6B). Importantly, overexpression of CD44 abrogated the restoration of CTX responsiveness by miR-302a overexpression in Caco-2 cells (Figure 6C). These results indicate that CD44 suppression sensitizes CRC cells to CTX treatment.

Increasing evidence indicates that CSCs are enriched and activated during cancer therapies. As a CSC marker, CD44 is thought to be essential for the drug resistance of CSCs by activating stem cell regulatory genes and by supporting CSC maintenance 30-32. We then determined whether the effect of miR-302a on CTX resistance is mediated by inhibiting CSC-like properties in CRC cells. We performed tumor sphere formation assays using Caco-2 and HCT116 cells to enrich cells with CSC-like characteristics, and qPCR showed that expression of the stem cell markers SOX2, OCT4, Nanog and KLF4 was high in the tumor spheres (Figure S3C). miR-302a overexpression or CD44 knockdown reduced the number and diameter of tumor spheres and decreased stem cell marker expression (Figure 6D-G). Notably, the reduction was more pronounced in the presence of CTX (Figure 6D-E). Furthermore, ectopic CD44 expression abrogated the suppressive effect of miR-302a on the sphere-forming ability of CRC cells in the presence or absence of CTX (Figure 6H and Figure S3D). Compared with adherent cells, tumor sphere cells exhibited higher CD44 expression but showed no difference in NFIB expression (Figure S3E), suggesting that miR-302a suppressed the CSC-like phenotype by targeting CD44 but not NFIB.

CD44 has been reported to interact with EGFR and to promote the activation of EGFR downstream MAPK and AKT signaling 22, 33. We found that EGFR was downregulated when CD44 was silenced in SW1463 and CC-CR cells (Figure 6I and Figure S3F). The overexpression of miR-302a and the knockdown of CD44 decreased the total level of EGFR as well as the phosphorylation levels of EGFR, AKT and ERK1/2 (Figure 6J). Moreover, CD44 overexpression abrogated the EGFR downregulation induced by miR-302a overexpression (Figure S3G). These results suggest that the CD44-mediated EGFR expression and activation of MAPK and AKT pathways could be inhibited by miR-302a.

### Inverse correlations between miR-302a and its targets in human CRC specimens

**Figure 7:**
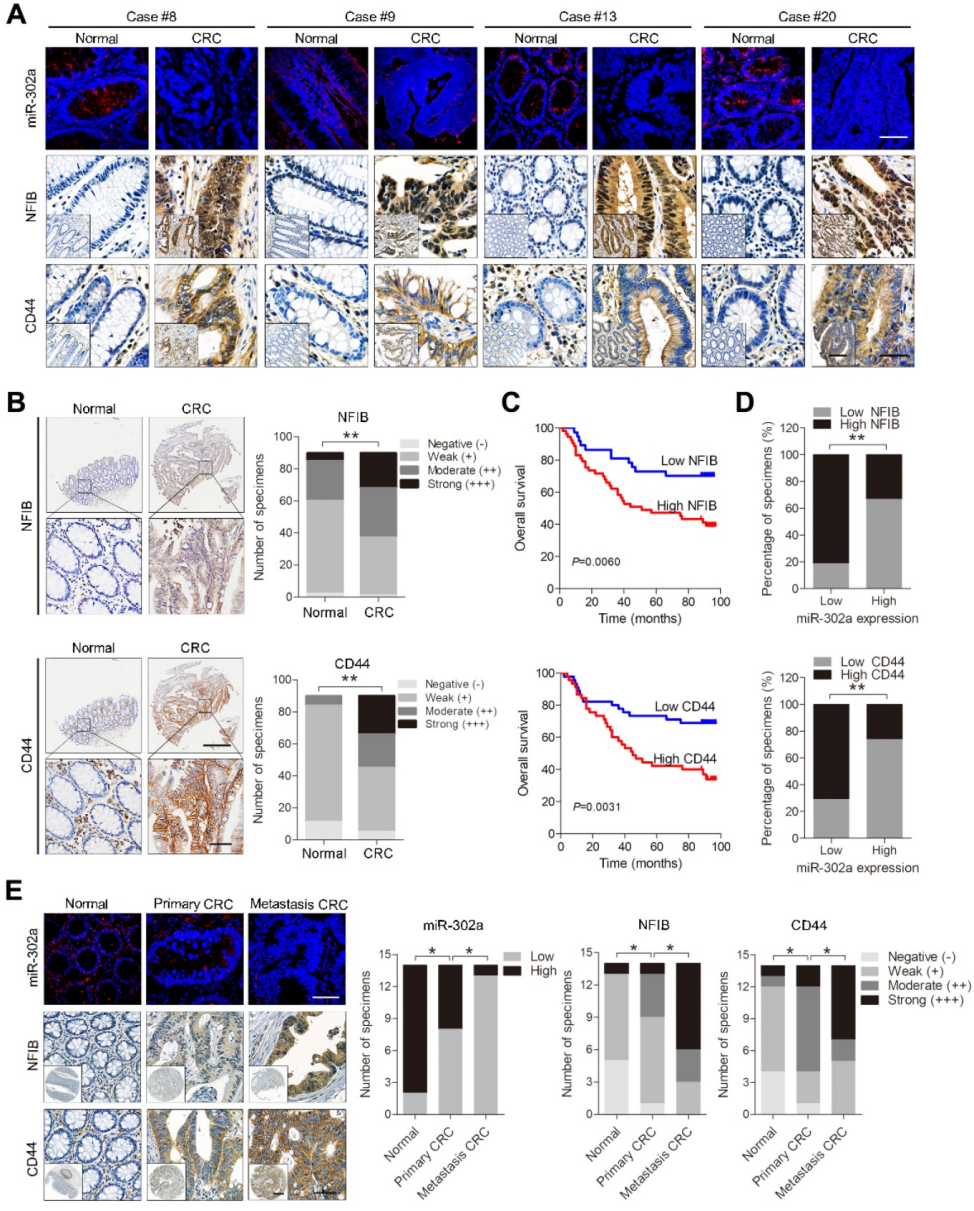
** Expression of miR-302a, NFIB and CD44 in human CRC tissues. A** Representative FISH images of miR-302a and corresponding IHC staining images of NFIB and CD44 from the 20 paired human CRC specimens and their adjacent normal tissues. Scale bar, 50 μm (FISH assays), 200 μm (inset) and 50 μm (main). **B** Representative images and analysis of IHC staining for NFIB and CD44 in 90 paired CRC specimens and their adjacent normal tissues. Scale bar, 500 μm (top) and 50 μm (bottom). **P<0.01, χ^2^ test. **C** Kaplan-Meier analysis of overall survival of CRC patients (n=90) according to high or low expression of NFIB and CD44. Significance determined by log-rank test. **D** The associations between miR-302a expression and NFIB or CD44 levels in 90 paired CRC specimens. **P<0.01, Spearman correlation. **E** Representative FISH images of miR-302a and corresponding IHC staining images of NFIB and CD44 in 14 pairs of primary CRC tissue and metastatic tissue. Scale bar, 50 μm (FISH assay), 500 μm (inset) and 50 μm (main). *P<0.05, χ^2^ test.

To evaluate the clinical significance of miR-302a, we detected miR-302a by FISH and NFIB and CD44 by IHC staining in 20 paired human CRC tissues and normal adjacent tissues previously used for miR-302a qPCR detection. miR-302a expression was decreased in most cancer tissues, which is consistent with the qPCR results, and NFIB and CD44 expression was increased in the corresponding cancer tissues compared with the matched adjacent normal tissues (Figure 7A and Figure S4A). In addition, miR-302a expression presented an inverse correlation with NFIB staining (*P*=0.0216), but the correlation between miR-302a expression and CD44 staining did not reach statistical significance (*P*=0.0622, Figure S4B). We further performed IHC staining for NFIB and CD44 on TMA 1. NFIB and CD44 expression was increased in most CRC tissues compared with adjacent normal tissues (Figure 7B).

Kaplan-Meier survival analysis indicated that high NFIB or CD44 expression was associated with shorter overall survival than low NFIB or CD44 expression (Figure 7C). Notably, a negative correlation between miR-302a expression and NFIB or CD44 expression was observed (Figure 7D). Since crosstalk exists between metastasis and drug resistance 5-9, we further detected the expression of miR-302a, NFIB and CD44 in a metastatic tissue microarray (TMA 2), which contained paired normal colon tissue, primary CRC tissue and distant metastatic tissue samples from 14 cases. The results show that miR-302a expression was decreased in CRC tissues compared with adjacent normal tissues and was further decreased in metastatic tissues, while the expression of NFIB and CD44 was increased in CRC tissues compared with adjacent normal tissues and was further increased in metastatic tissues (Figure 7E). In addition, we found that ITGA6 expression was also increased in CRC tissues compared with adjacent normal tissues (Figure S4C). High ITGA6 expression was associated with shorter overall survival than low ITGA6 expression (Figure S4D). ITGA6 expression was positively correlated with NFIB expression but negatively correlated with miR-302a expression in CRC tissues (Figure S4E).

## Discussion

The coding gene of miR-302a is located in the 4q25 region of human chromosome 4, which is conserved in vertebrates based on a comparison of homologous sequences 34. miR-302a can regulate certain developmental processes, such as neuronal differentiation and stem cell self-renewal 35, 36. miR-302a has been characterized as a tumor suppressor in several cancer types, including breast cancer 37, liver cancer 13 and glioma 12. However, it has also been reported to promote the development and progression of certain cancers, such as pancreatic cancer 38 and prostate cancer 39, suggesting a context-dependent role of miR-302a in different tissues. However, some studies have provided contradictory data even within the same cancer type. Although studies have reported reduced miR-302a expression in CRC 14, other research found increased miR-302a expression in colonic cancerous tissues compared with normal mucosa 16. This discrepancy may be caused by challenges in acquiring patient samples at equivalent stages of disease and in classifying the disease state based on different methodologies. In the present study, we determined the expression pattern and function of miR-302a and provided several lines of evidence indicating that miR-302a acts as a tumor suppressor in CRC. We found that miR-302a expression was decreased in most CRC cell lines and specimens. Importantly, reduced miR-302a expression in cancerous specimens predicted poor prognosis in CRC patients. miR-302a expression was also found to be decreased in CTX-resistant CRC cells, which is consistent with observations from CTX-resistant PDXs. Functionally, miR-302a overexpression inhibited CRC cell migration, invasion and metastasis. miR-302a overexpression also restored CTX responsiveness of CRC cells with wild-type *KRAS* and* BRAF* genes both *in vitro* and *in vivo*. These findings indicate that miR-302a expression is often decreased in CRC and that its overexpression could inhibit CRC cells metastasis and sensitize CRC cells with wild-type* KRAS*/*BRAF* to anti-EGFR therapy.

Multiple transcription factors have been reported to participate in the metastasis cascade 40. NFIB is a transcription factor that belongs to the nuclear factor I (NFI) family, which regulates development and cellular differentiation in several tissue types 41. Recently, increased copy numbers and NFIB overexpression have been found during tumorigenesis 42. A previous study revealed that NFIB promoted pro-metastatic neuronal gene expression by stabilizing chromatin accessibility in small cell lung cancer cells 21. However, little is known regarding the function and regulatory mechanism of NFIB in CRC. In the present study, NFIB was identified as a direct target of miR-302a that functions as a metastasis promoter in CRC. We confirmed that miR-302a directly binds to the 3'-UTR of NFIB, and the manipulation of miR-302a affected NFIB protein expression. We found that NFIB was often increased in CRC cell lines and tissues, and its overexpression predicted poor prognosis in CRC patients. Moreover, a negative correlation between miR-302a and NFIB was confirmed in a large-scale study of CRC tissues. NFIB was further identified as a functional target of miR-302a based on the finding that the metastasis-promoting effect of NFIB could be abrogated by miR-302a overexpression, which also suggests that the anti-metastatic role of miR-302a is mediated through NFIB suppression.

Integrins are heterodimeric integral membrane proteins composed of an α chain and a β chain that function in cell surface adhesion and signaling 43. Integrins have been implicated in nearly every step of cancer progression and metastasis 43. ITGA6 encodes a member of the integrin α chain family, which may associate with a β1 or β4 subunit to form an integrin 44. Previous studies have reported that ITGA6 overexpression enhances metastatic potential in several cancer types 25, 45, 46. In the present study, we identified ITGA6 as a transcriptional target of NFIB and demonstrated that silencing ITGA6 suppressed CRC cell migration and invasion. We also found that ITGA6 expression was often increased in CRC tissues, and ITGA6 overexpression predicted poor prognosis in CRC patients. Moreover, we revealed that ITGA6 expression was positively correlated with NFIB expression but negatively correlated with miR-302a expression in CRC specimens. These results suggest that miR-302a may target the NFIB/ITGA6 axis in CRC.

Current studies indicate that CD44 is an important marker for CSCs that contributes to the activation of stem cell regulatory genes and supports the maintenance of CSCs 30-32. Ample evidence indicates that CSCs are enriched or activated during cancer therapies, and CD44 contributes to the drug resistance of CSCs 30, 47, 48. For instance, CD44^high^ breast cancer cells that survive epirubicin treatment exhibit the growth and gene expression signatures of CSCs 49. In the present study, we identified CD44 as a direct and functional target of miR-302a in the regulation of CTX resistance in CRC. We found that CD44 silencing sensitized CRC cells to CTX treatment, while CD44 overexpression induced CTX resistance, but this effect could be abrogated by the ectopic expression of miR-302a. We also found that CD44 was upregulated in stem cell-enriched tumor spheres, and miR-302a overexpression or CD44 silencing showed consistent inhibitory effects on tumor sphere growth and the expression of stem cell markers. In addition, we observed a negative correlation between miR-302a and CD44 in CRC tissues. These results suggest that miR-302a regulates the CTX resistance of CRC cells possibly though the suppression of CD44 and its effects on CSCs.

EGFR-mediated signaling via the Ras-MAPK and PI3K-AKT pathways requires the CD44 cytoplasmic tail associated with receptor tyrosine kinases 22, 30, 33, 47. The upregulation of CD44 and its variant isoforms promotes the activation of EGFR downstream signaling pathways and consequently leads to tumor cell migration and drug resistance 22, 50. In the present study, we showed that CD44 suppression inhibited EGFR expression and the activation of its downstream signaling pathways in CRC cells. These effects are consistent with those of miR-302 overexpression, suggesting that the tumor suppressive role of miR-302a might be partially mediated by targeting the CD44/EGFR axis in CRC.

High frequencies of metastasis and drug resistance are the main obstacles leading to a poor prognosis in CRC patients. Therefore, determination of the regulatory mechanism of CRC metastasis and drug resistance and the development of novel therapies are urgently needed. This study investigated the roles and functional mechanisms of miR-302a in CRC. We found that miR-302a inhibited metastasis by decreasing activity of the NFIB/ITGA6 axis and sensitized CRC cells to CTX by suppressing the CD44-mediated CSC-like properties and EGFR downstream signaling. These results suggest that the restoration of miR-302a expression might be a potential therapeutic strategy for halting CRC progression.

## Supplementary Material

Supplementary figures, tables 1, 3, 4, 5, 6.

Supplementary table 2.

Supplementary table 7.

## Abbreviations

CRC: colorectal cancer
CTX: cetuximab
NCCN: National Comprehensive Cancer Network
miRNA: microRNA
3D: three-dimensional
PDX: patient-derived xenograft
NFIB: nuclear factor I B
ITGA6: integrin subunit alpha 6
CSC: cancer stem cell
PFA: paraformaldehyde
FFPE: formalin-fixed paraffin-embedded
H&E: hematoxylin and eosin
ChIP: chromatin immunoprecipitation
IF: immunofluorescence
FISH: fluorescence *in situ* hybridization
IC50: 50% inhibitory concentration
DIG: digoxigenin
TSA: tyramide signal amplification
DAB: diaminobenzidine tetrahydrochloride
IHC: immunohistochemical
