# Supplementary material for: miR-302a Inhibits Metastasis and Cetuximab Resistance in Colorectal Cancer by Targeting NFIB and CD44
Source: Theranostics. 2019 Oct 22;9(26):8409–25. doi: 10.7150/thno.36605 (PMC6857048; doi:10.7150/thno.36605)
Supplement: Supplementary file 1 — Supplementary figures, tables 1, 3, 4, 5, 6. [file thnov09p8409s1.pdf]

Supplementary Figures

Figure S1

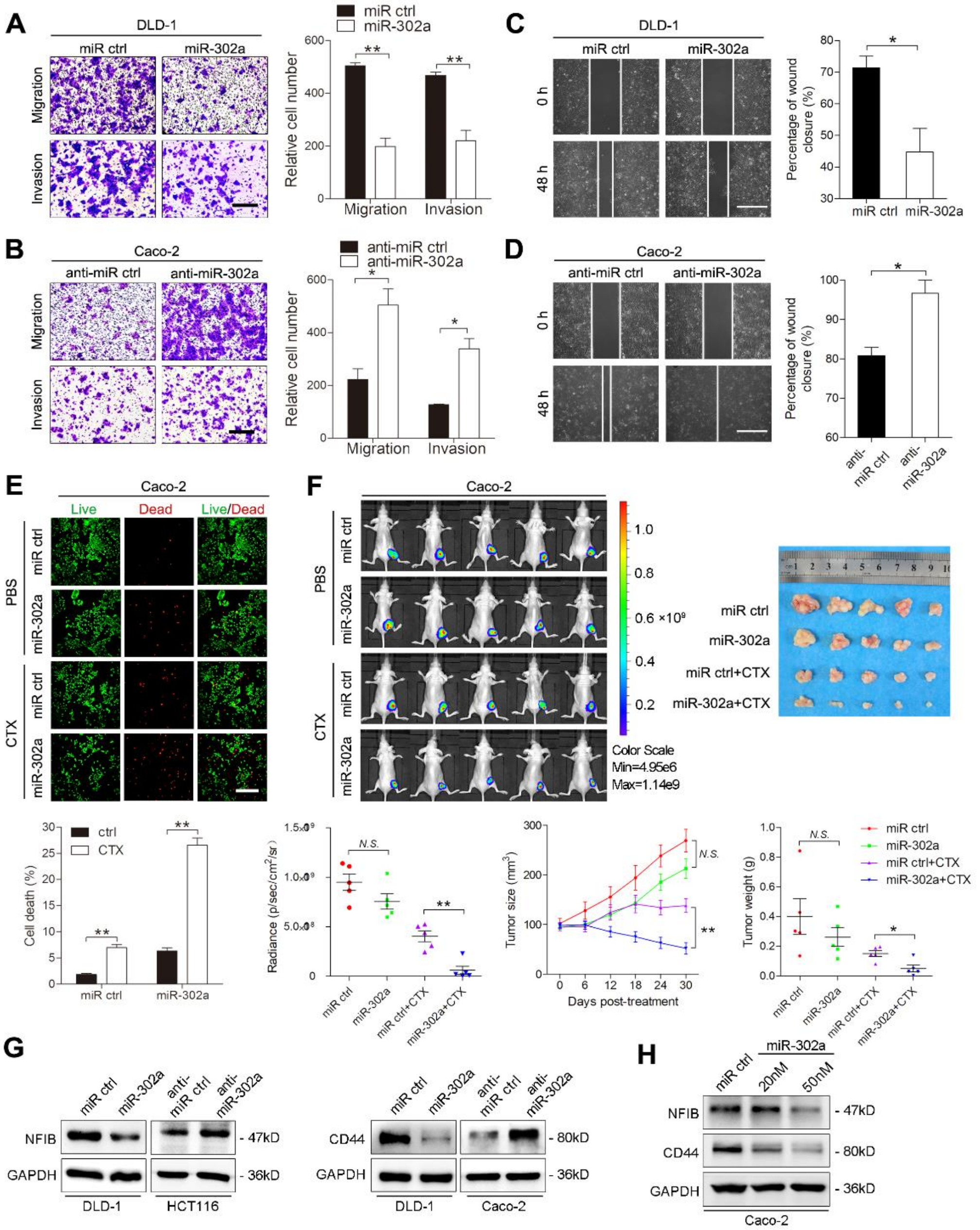

Figure S1 miR-302a inhibits migration and invasion *in vitro* and restores CTX responsiveness *in vivo* by targeting NFIB and CD44. A, B Representative images and graph of up- and downregulation of miR-302a in DLD-1 (A) and Caco-2 (B) cells, as determined by Transwell migration and invasion assays. Scale bar, 500 μm. **C, D** Representative images and graph of up- and downregulation of miR-302a in DLD-1 (C) and Caco-2 (D) cells, as determined by wound-healing assay. Scale bar, 500 μm. **E** Representative images of Caco-2 cells after fluorescence staining for LIVE/DEAD cell viability assay. Green: active esterase in the cytoplasm stained with calcein AM (live cells); red: damaged cell membrane allows ethidium homodimer to bind to DNA (dead cells). Images were acquired 72 h after CTX treatment. Scale bar, 500 μm. **F** Bioluminescent images, anatomical photos of tumors, and graph of tumor weights and volumes of xenografts treated with CTX from mice implanted with control or miR-302a-overexpressing Caco-2 cells (n=5). **G, H** Western blot analysis of NFIB and CD44 expression with miR-302a up- and downregulation in the indicated cells. The values represent the mean ± SEM. *P<0.05, **P<0.01, *N.S.*, not significant.

Figure S2

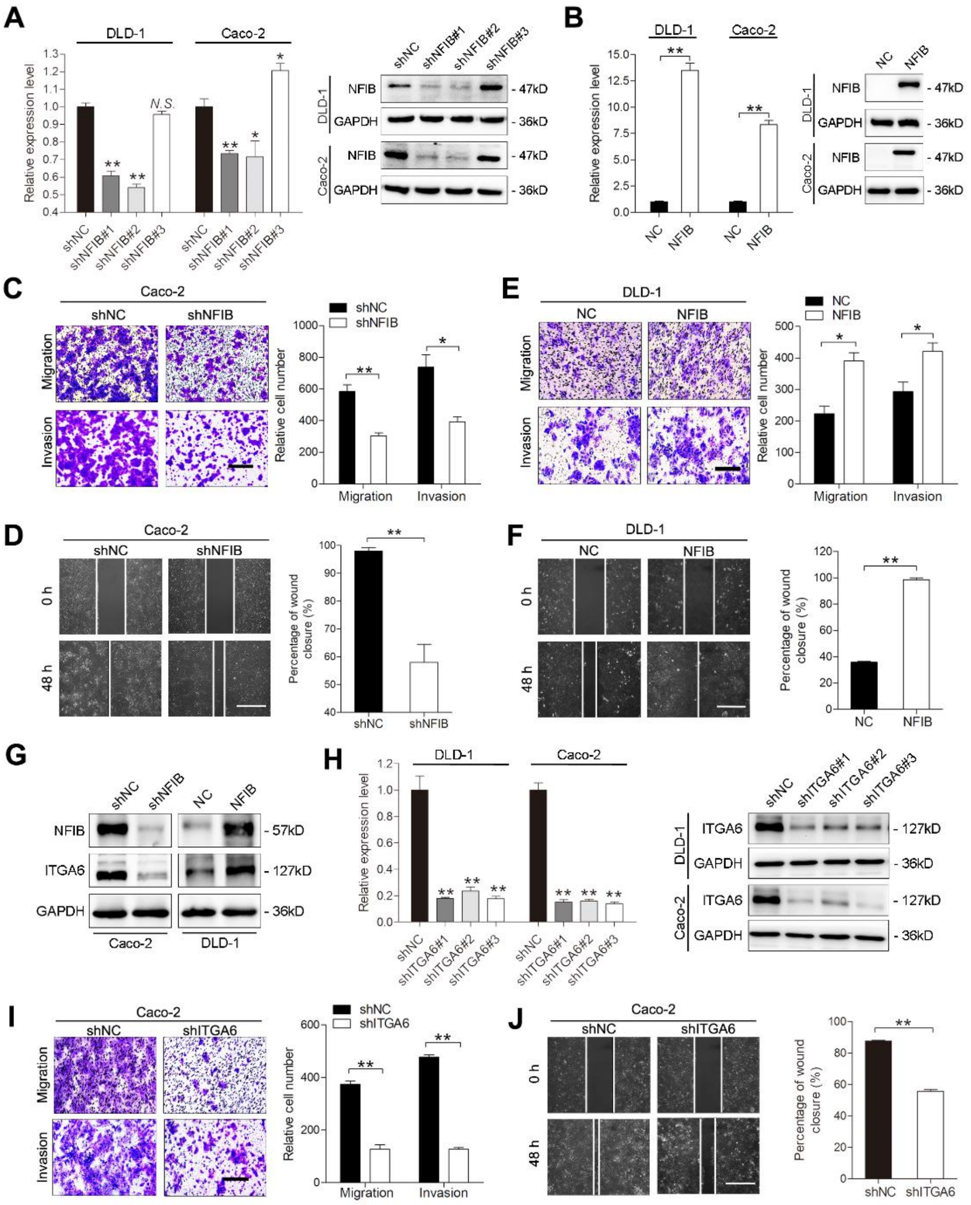

Figure S2 NFIB and ITGA6 promotes the migration and invasion of CRC cells. A qPCR and Western blot analyses of NFIB expression in DLD-1 and Caco-2 cells infected with a specific shRNA for NFIB (shNFIB) or a negative control (shNC). **B** qPCR and Western blot analyses of NFIB expression in DLD-1 and Caco-2 cells infected with lentiviral NFIB or a negative control (NC). **C, D** Representative images and graph of NFIB up- or downregulation in Caco-2 cells, as determined by Transwell migration and invasion assays (C) and wound-healing assay (D). Scale bar, 500 µm. **E, F** Representative images and graph of NFIB up- or downregulation in DLD-1 cells, as determined by Transwell migration and invasion assays (E) and wound-healing assay (F). Scale bar, 500 µm. **G** Western blot analyses of ITGA6 expression with NFIB down- and upregulation in Caco-2 and DLD-1 cells. **H** qPCR and Western blot analyses of ITGA6 expression in DLD and Caco-2 cells infected with a specific shRNA for ITGA6 (shITGA6) or a negative control (shNC). **I, J** Representative images and graph of ITGA6 up- or downregulation in Caco-2 cells, as determined by Transwell migration and invasion assays (I) and wound-healing assay (J). Scale bar, 500 µm. The values represent the mean ± SEM. **P*<0.05, ***P*<0.01, *N.S.*, not significant.

Figure S3

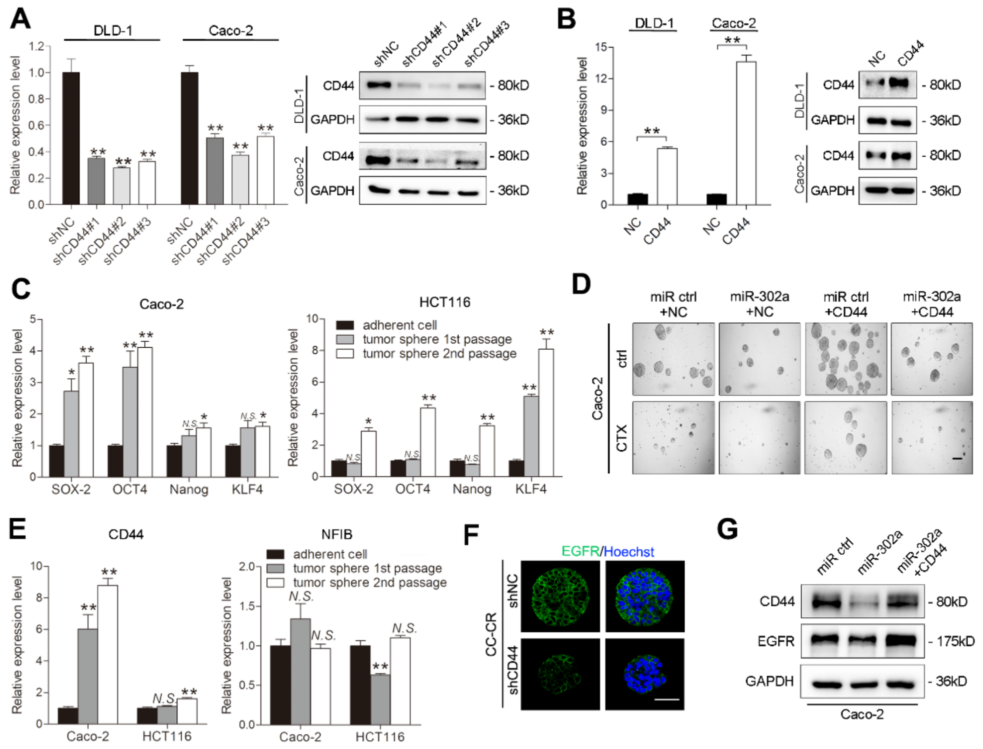

Figure S3 miR-302a suppresses CSC-like characteristics and EGFR expression in CTX treatment by suppressing CD44 in CRC cells. A qPCR and Western blot analyses of CD44 expression in DLD-1 and Caco-2 cells infected with a specific shRNA for CD44 (shCD44) or a negative control (shNC). **B** qPCR and Western blot analyses of CD44 expression in DLD-1 and Caco-2 cells infected with lentiviral CD44 or a negative control (NC). **C** qPCR analyses of SOX-2, OCT4, Nanog and KLF4 expression in the indicated cells. **D** Representative micrographs of tumor spheres formed when transfected with miR-302a and/or CD44 in Caco-2 cells in the presence or absence of CTX (100 μg/ml). Scale bar, 200 μm. **E** qPCR analyses of CD44 and NFIB expression in the indicated cells. **F** Representative immunofluorescence images of EGFR staining with CD44 downregulation in CC-CR cells in a 3D culture system. Scale bar, 50 μm. **G** Western blot analyses of CD44 and EGFR expression when transfected with miR-302a and/or CD44 in Caco-2 cells. The values represent the mean ± SEM. **P*<0.05, ***P*<0.01, *N.S.*, not significant.

Figure S4

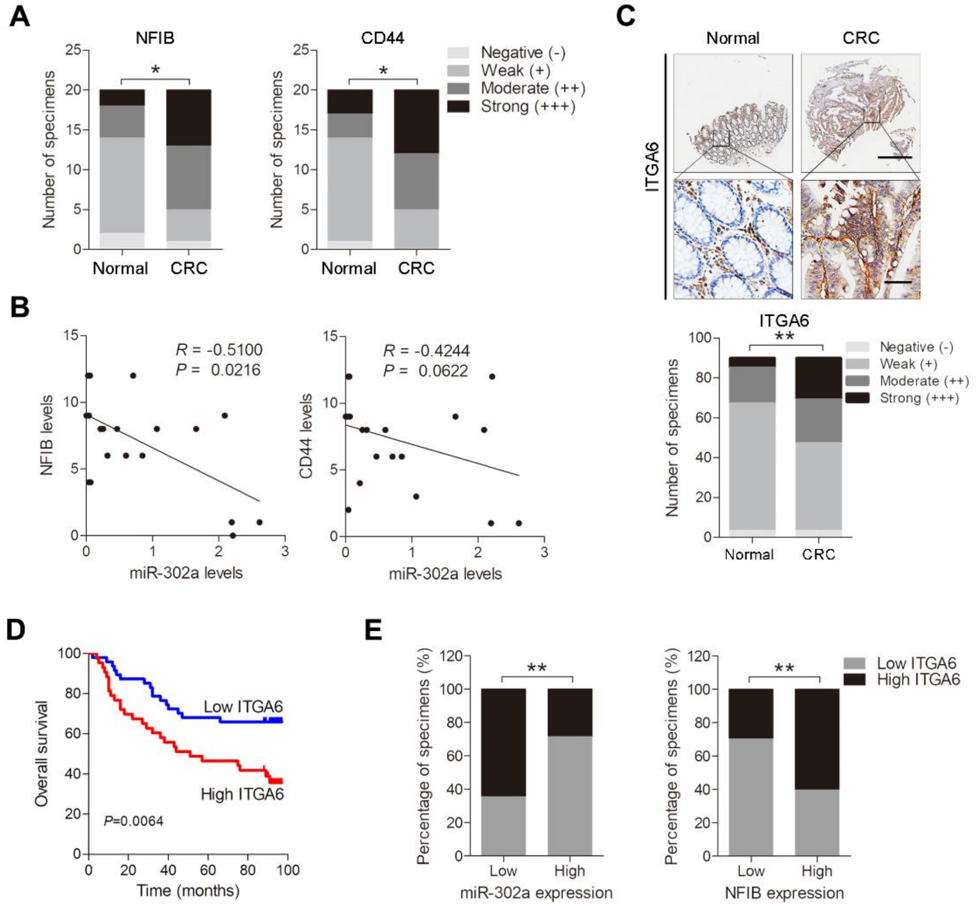

Figure S4 Expression of miR-302a, NFIB, CD44 and ITGA6 in human CRC tissues. A Analysis of IHC staining of NFIB and CD44 in 20 paired human CRC specimens and their adjacent normal tissues. **B** Correlations between miR-302a and NFIB or CD44 levels in the 20 paired human CRC specimens and their adjacent normal tissues. Significance determined by Spearman correlation. **C** Representative images and analysis of IHC staining for ITGA6 expression in 90 paired CRC specimens and their adjacent normal tissues. Scale bar, 500 μm (top) and 50 μm (bottom). **D** Kaplan–Meier analysis of overall survival of CRC patients according to high or low ITGA6 expression. **E** Associations between ITGA6 and miR-302a or NFIB levels in the 90 paired CRC specimens. **P*<0.05, ***P*<0.01.

Supplementary Table 1 Origin and genetic information of cell lines used in this study

Cell line	Disease	Tissue	Mutational status
HIEC	Normal	Small intestine	WT
HCT116	Colorectal carcinoma	Colon	KRAS
DiFi	Familial adenomatous polyposis	Rectum	WT
HCT8	Colorectal adenocarcinoma	Ileocecal colon	KRAS
HT29	Colorectal adenocarcinoma	Colon	BRAF
LoVo	Colorectal adenocarcinoma	Metastatic site: left supraclavicular region	KRAS
RKO	Colorectal carcinoma	Colon	BRAF
DLD-1	Colorectal carcinoma	Colon	KRAS
SW620	Colorectal adenocarcinoma	Metastatic site: lymph node	KRAS
SW480	Colorectal adenocarcinoma	Colon	KRAS
Caco-2	Colorectal adenocarcinoma	Colon	WT
SW1463	Colorectal adenocarcinoma	Rectum	KRAS
T84	Colorectal carcinoma	Metastatic site: lung	KRAS
SW948	Colorectal adenocarcinoma	Colon	KRAS

Mutational status refers to the status of KRAS and BRAF genes.

Supplementary Table 3 shRNA sequence used in this study

shRNA	Sense (5' to 3')	Antisense (5' to 3')
shNC	UUCUCCGAACGUGUCACGUTT	ACGUGACACGUUCGGAGAATT
shNFIB#1	GAUGUAUUCUCCCAUCUGUTT	ACAGAUGGGAGAAUACAUCTT
shNFIB#2	GGAGUUGCACACAGUGUCAUCUCAA	UUGAGAUGACACUGUGUGCAACUCC
shNFIB#3	CCUUCCUACAUCAGCAACATT	UGUUGCUGAUGUAGGAAGGTT
shITGA6#1	GGUGGCAAGAUAUAGUUAUTT	AUAACUAUAUCUUGCCACCTT
shITGA6#2	CAGGUUCUCAAGGGUAUAUTT	AUAUACCCUUGAGAACCUGTT
shITGA6#3	GGCCUGUGAUUAAUAUUCATT	UGAAUAUUAAUCACAGGCCTT
shCD44#1	CUCCCAGUAUGACACAUAUTT	AUAUGUGUCAUACUGGGAGTT
shCD44#2	GGACCAAUUACCAUAACUATT	UAGUUAUGGUAAUUGGUCCTT
shCD44#3	GCAGUCAACAGUCGAAGAATT	UUCUUCGACUGUUGACUGCTT

Supplementary Table 4 Primers used in this study

Primer	Forward sequence (5' to 3')	Reverse sequence (5' to 3')
GAPDH	GCACCGTCAAGGCTGAGAAC	TGGTGAAGACGCCAGTGGA
NFIB	CTTATCCAATCCCGACCAGA	GACTAGATCCAGACGCCAGACT
ITGA6	TTGTTGGCGAGCAAGCTATGA	TTGCTGTGCCGAGGTTTGTAA
CD44	GCATTGCAGTCAACAGTCGAAGA	CCTTGTTCACCAAATGCACCA
EGFR	TGATAGACGCAGATAGTCGCC	TCAGGGC ACGGTAGAAGTTG
SOX-2	CCAAGATGCACAACTCGGAGA	CCGGTATTTATAATCCGGGTGCT
OCT4	GTGCCGTGAAGCTGGAGAA	TGGTCGTTTGGCTGAATACCTT
Nanog	CCTGTGATTTGTGGGCCTGA	CTCTGCAGAAGTGGGTTGTTTG
KLF4	AAGAGTTCCCATCTCAAGGCACA	GGGCGAATTTCCATCCACAG
Epcam	AAGGACACTGAAATAACCTGCTCTG	TTGATAACGCGTTGTGATCTCC
CD133	AGTGGCATCGTGCAAACCTG	CTCCGAATCCATTCGACGATA
CD166	CATTATCATACCTTGCCGACTTG	TGTATTCTGGTACATCGTCGTACTG
NFIB miR-302a binding site 1 mutagenesis primer	AAGAAGATAATAGACCAGCAATTGCCATCTGGCCAATCACTAATTCCCTTAAGGTTGA	TCAACCTTAAGGGAATTAGTGATTGGCCAGATGGCAATTGCTGGTCTATTATCTTCTT
NFIB miR-302a binding site 2 mutagenesis primer	AGAAAAATAAAATTAATGAAAACACCATCTGGCTGTTGGTTTAGCTGCAGCCTCCTTG	CAAGGAGGCTGCAGCTAAACCAACAGCCAGATGGTGTTTTCATTAATTTTATTTTTCT

CD44 miR-302a binding site mutagenesis primer	GCTGAGTTGAACATCTGGATTGGAAAATATTAAAA GGCTAACATTAAAAGACTAAAGGAAACAGA	TCTGTTTCCTTTAGTCTTTTAATGTTAGCCTTTTAA TATTTTCCAATCCAGATGTTCAACTCAGC
ITGA6 ChIP1 primer	ATAGTTAACAGCTGGTGTGAC	TCCAAAGTGGGGTGCAGTGG
ITGA6 ChIP2 primer	GCTCCTGCTCTTTCCTGGGGC	TGGTTTTCAGCAGCCTGCCAA
ITGA6 ChIP3 primer	CTAAATGTCAGAGGTTGGTG	CGATGCCGTTCTTTCCTAGCC
ITGA6 ChIP4 primer	TAGGTGATCTGGGGACAAGGC	AGATGTGGGCCCACGTGCAG
ITGA6 ChIP control primer	TAGGCCAGGGGAGGTGGCTC	TACAGGCACATGCCACCATGC

Supplementary Table 5 Primary antibodies used in this study

Antibody	Company	Catalog No.	Application	Dilution
GAPDH	Proteintech	10494-1-AP	Western blot	1:2,000
NFIB	Abcam	ab186738	Western blot/ChIP	1:1,000
NFIB	Sigma-Aldrich	HPA003956	IHC staining	1:500
ITGA6	Cell Signaling Technology	37500	Western blot	1:1,000
ITGA6	Abcam	ab181551	IHC staining	1:500
CD44	Cell Signaling Technology	3570	Western blot	1:1,000
CD44	Abcam	ab157107	IHC staining	1:2,000
CD44	BioLegend	103011	IF	1:100
EGFR	Cell Signaling Technology	4267	Western blot	1:1,000
EGFR	Cell Signaling Technology	4267	IF	1:50
p-EGFR	Cell Signaling Technology	3777	Western blot	1:1,000
AKT	Cell Signaling Technology	4691	Western blot	1:1,000
p-AKT	Cell Signaling Technology	4060	Western blot	1:1,000
ERK1/2	Cell Signaling Technology	4695	Western blot	1:1,000
p-ERK1/2	Cell Signaling Technology	4370	Western blot	1:1,000
Ki-67	Cell Signaling Technology	9449	IHC staining	1:800
Cleaved Caspase-3	Cell Signaling Technology	9664	IHC staining	1:400

Supplementary Table 6 The relationship of clinicopathological factors and the miR-302a, NFIB, CD44 and ITGA6 expression in the tissue microarray

	All	miR-302a			NFIB					CD44					ITGA6				
	cases	Low	High	*P*	-	+	++	+++	*P*	-	+	++	+++	*P*	-	+	++	+++	*P*
**Gender**				0.6726					0.4947					0.8782					0.7274
Female	45	23	22		0	17	16	12		1	21	10	13		1	20	12	12	
Male	45	25	20		1	22	12	10		4	21	11	9		2	24	10	9	
**Age (years)**				0.4785					0.5089					0.5507					0.6268
≤65	40	23	17		0	15	15	10		3	16	9	12		2	17	10	11	
＞65	50	25	25		1	24	13	12		2	26	12	10		1	27	12	10	
**Tumor size (cm)**				0.0028					0.0659					0.0336					0.032
≤5	56	23	33		1	28	16	11		4	28	15	9		2	34	10	10	
＞5	34	25	9		0	8	15	11		1	12	6	15		1	10	12	11	
**Differentiation**				0.0068					0.0009					0.0018					0.0059
High	12	4	8		0	9	1	2		0	10	2	0		1	7	3	1	
Moderate	69	35	34		1	27	28	13		5	29	18	17		2	36	18	13	
Poor	9	9	0		0	0	2	7		0	1	1	7		0	1	1	7	
**TNM stage**				0.0446					0.0005					0.0341					0.0221
I	6	2	4		0	4	2	0		1	3	1	1		0	2	4	0	
II	49	21	28		0	27	5	11		3	29	9	8		2	31	9	7	
III	34	24	10		1	5	17	11		1	8	10	15		1	11	9	13	
IV	1	1	0		0	0	1	0		0	0	1	0		0	0	0	1	
